# Occurrence of tick-borne pathogens in dogs in a coastal region of the state of Ceará, northeastern Brazil

**DOI:** 10.1590/S1984-29612022010

**Published:** 2022-02-28

**Authors:** Arícia Débora Vasconcelos Fonsêca, Lorena Mayana Beserra de Oliveira, Felipe Rodrigues Jorge, Ramuelly Olinda Cavalcante, Claudia Maria Leal Bevilaqua, Francisco José Maia Pinto, Jessica Maria Leite dos Santos, Bruno Marques Teixeira, Ana Kétylla Ponte Prado Rodrigues, Gissandra Farias Braz, Geysa Almeida Viana, Edmara Chaves Costa, Maria Carolina de Azevedo Serpa, Bárbara Conte Weck, Marcelo Bahia Labruna

**Affiliations:** 1 Laboratório de Doenças Parasitárias, Programa de Pós-graduação em Ciências Veterinárias, Universidade Estadual do Ceará – UECE, Fortaleza, CE, Brasil; 2 Centro de Ciências da Saúde, Universidade Estadual do Ceará – UECE, Fortaleza, CE, Brasil; 3 Centro de Ciências da Saúde, Centro Universitário INTA – UNINTA, Sobral, CE, Brasil; 4 Instituto de Ciências da Saúde, Universidade da Integração Internacional da Lusofonia Afro-Brasileira – UNILAB, Redenção, CE, Brasil; 5 Departamento de Medicina Veterinária Preventiva e Saúde Animal, Faculdade de Medicina Veterinária e Zootecnia, Universidade de São Paulo – USP, São Paulo, SP, Brasil

**Keywords:** Babesia vogeli, Hepatozoon canis, Ehrlichia canis, *Rickettsia* spp., epidemiology, Babesia vogeli, Hepatozoon canis, Ehrlichia canis, *Rickettsia* spp., epidemiologia

## Abstract

The aim of this study was to determine the occurrence of tick-borne pathogens (*Ehrlichia canis*, *Babesia vogeli*, *Hepatozoon* spp. and *Rickettsia* spp.) in dogs in Vila de Jericoacoara, coastal region of Ceará, Brazil. Blood samples were collected from 153 animals and analyzed using molecular and serological methods. Sixty animals were found to be infected or exposed to at least one of the pathogens studied. *Babesia vogeli* was the most prevalent pathogen (15%), followed by *E. canis* (13.7%) and *Hepatozoon* spp. (11.8%), which was identified as *Hepatozoon canis* through sequencing. Twenty dogs (13%) were seroreactive to *Rickettsia* spp. *Rhipicephalus sanguineus sensu lato* was observed on 11.8% of the animals. There were associations between age (< 3 years old) and positivity for *B. vogeli,* and between habitation (stray dogs) and positivity for *H. canis*. There were also associations between anemia and infection with *H. canis*, and between leukopenia and exposure to *Rickettsia* spp. No association was detected between clinical alterations and infection with or exposure to the pathogens studied. The results confirmed that pathogens of veterinary importance are circulating in northeastern Brazil and showed that dogs are exposed to *Rickettsia* species with zoonotic potential, thus indicating a need for vector control measures.

## Resumo

O objetivo deste estudo foi determinar a ocorrência de patógenos transmitidos por carrapatos (*Ehrlichia canis*, *Babesia vogeli*, *Hepatozoon* spp. e *Rickettsia* spp.) em cães na Vila de Jericoacoara, região costeira do Ceará, Brasil. Amostras de sangue foram coletadas de 153 animais e analisadas por métodos moleculares e sorológicos. Sessenta animais foram encontrados infectados ou expostos a pelo menos a um dos patógenos estudados. *Babesia vogeli* foi o patógeno mais prevalente (15%), seguido por *E. canis* (13,7%) e *Hepatozoon* spp. (11,8%), que foi identificado como *Hepatozoon canis* por sequenciamento. Vinte cães (13%) foram sororreativos à *Rickettsia* spp. *Rhipicephalus sanguineus sensu lato* foi observado em 11,8% dos animais. Houve associações entre idade (<3 anos) e positividade para *B. vogeli*, e entre habitação (cães de rua) e positividade para *H. canis*. Também houve associações entre anemia e infecção por *H. canis*, e entre leucopenia e exposição a *Rickettsia* spp. Não foi detectada associação entre alterações clínicas e infecção ou exposição aos patógenos estudados. Os resultados confirmaram que patógenos de importância veterinária estão circulando no nordeste do Brasil e mostraram que cães estão expostos a espécies de *Rickettsia* com potencial zoonótico, indicando a necessidade de medidas de controle do vetor.

## Introduction

The emergence and reemergence of arthropod-borne diseases has been a challenge for veterinary and human medicine. Arthropods and the infections transmitted by them are expanding their zoogeographical limits due to climate change and increased accessibility to certain environmental niches (Shaw et al., 2001; Han et al., 2016). Common species of tick-borne pathogens include *Babesia vogeli*, *Hepatozoon canis*, *Ehrlichia canis* and *Rickettsia* spp. of the spotted fever group (SFG). These pathogens cause canine diseases in several geographical regions including tropical areas (Chomel, 2011).

The above-cited tick-borne pathogens can be divided into two groups. The first includes the protozoa *B. vogeli* and *H. canis*, and the second includes the bacteria *E. canis* and *Rickettsia* spp. *Babesia vogeli* has worldwide distribution and usually gives rise to subclinical infection in adult domestic dogs, although it is potentially fatal in young dogs (Schnittger et al., 2012). *Hepatozoon canis* is distributed throughout the Old World and in parts of the New World, and domestic dogs infected with this agent present lethargy with mild anemia (Baneth, 2011). *Ehrlichia canis* is a common pathogen affecting domestic dogs around the world and causes canine monocytic ehrlichiosis, with clinical and hematological abnormalities such as fever, anorexia, vomiting, diarrhea, petechial hemorrhages, anemia and thrombocytopenia (Moreira et al., 2003; Moraes-Filho et al., 2015). Spotted fever group (SFG) rickettsiae are a neglected group of bacteria belonging to the genus *Rickettsia*, which accounts for a large number of new and emerging infectious diseases with worldwide distribution and can cause serious diseases in humans and animals (Labruna et al., 2009; Oliveira et al., 2016; Robinson et al., 2019).

In many parts of Brazil, there are records of dogs infected by tick-borne pathogens at wide ranges of occurrence and prevalence rates (Saito et al., 2008; Ramos et al., 2010; Spolidorio et al., 2011; Vieira et al., 2011; Costa et al., 2015; Miranda et al., 2014; Rotondano et al., 2015). In contrast, there is a scarcity of data about the epidemiology of tick-borne diseases in the coastal region of northeastern Brazil. Therefore, the aim of the present study was to make the first determination of occurrence rates of *B. vogeli*, *Hepatozoon* spp., *E. canis* and *Rickettsia* spp. in dogs and their ectoparasites in the municipality of Jijoca de Jericoacoara, located in the coastal region of the state of Ceará, Brazil. Furthermore, this study also investigated the possible epidemiological, clinical and hematological aspects of the diseases caused by these pathogens.

## Materials and Methods

### Ethics committee

The present cross-sectional, descriptive and analytical study was approved by the Ethics Committee on Animal Experimentation at Centro Universitário Inta (UNINTA), state of Ceará, Brazil (protocol number: 2019.07.009-P).

### Study area

**Figure 1:**
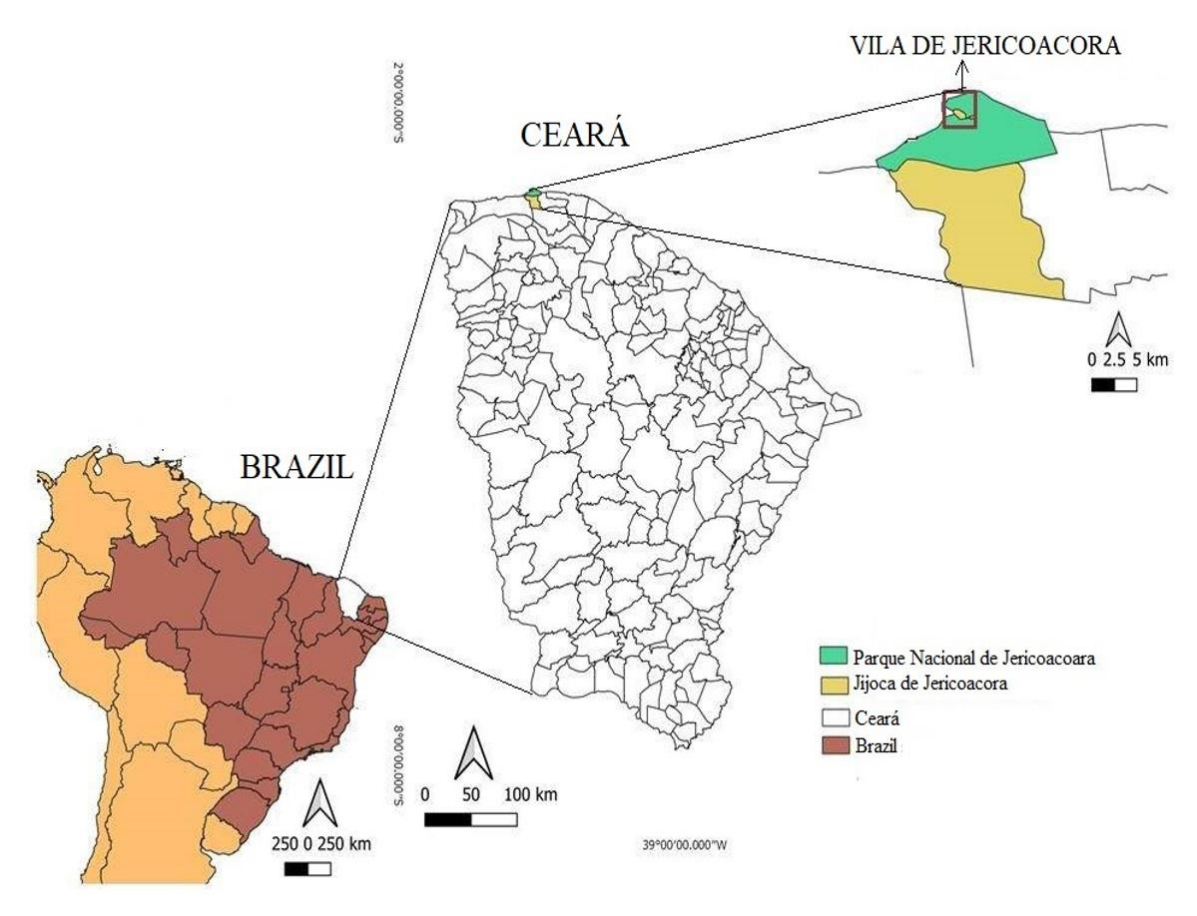
Geographical location of Vila de Jericoacoara, Jijoca de Jericoacoara, Ceará, Brazil.

This study was conducted in Vila de Jericoacoara (2° 47' 45” S; 40° 30' 52” W), which is a village within the municipality of Jijoca de Jericoacoara, state of Ceará (Figure 1), northeastern Brazil. This village is on the shore of the Atlantic Ocean and its other geographical limit is the National Park (PARNA) of Jericoacoara, a conservation unit that has the aims of protecting biodiversity and coastal ecosystems, ensuring the preservation of its natural resources and enabling scientific research, environmental education and ecological tourism (ICMBio, 2021). Vila de Jericoacoara covers an area of 1.1 km^2^ and its streets are unpaved. Its average annual temperature is 25-35 °C and it lies within the Caatinga biome, with the vegetation complex of the coastal zone.

### Animals

For this study, dogs living in Vila de Jericoacoara were selected through convenience sampling. This sample totaled 153 animals of both sexes and different ages and breeds. The dogs thus selected participated in an extension project carried out by a trained team from the Centro Universitário INTA (UNINTA) and by the association “Jeri sobre Patas” (Jericoacoara on Paws), between April 2019 and December 2020. This project was aimed towards population management of dogs and cats in the village. The owners of these dogs were made aware of the study objectives and, after agreeing to participate, signed informed consent statements.

### Data and sample collection

An epidemiological questionnaire was applied to each dog owner to obtain data for an analysis on factors associated with the outcome regarding the occurrence of infection or exposure to tick-borne pathogens. The possible variables considered in the questionnaire related to gender, breed, age, habitation, street access and presence of ectoparasites. In addition, each dog was physically examined for the presence of clinical signs suggestive of tick-borne diseases, including body condition, lymphadenopathy, weight loss, anorexia and fever. The dog owners were also asked about any recent episodes of vomiting and diarrhea. Body temperatures were measured using a digital thermometer.

Blood samples were collected from the jugular or cephalic vein of each animal. For hematological analyses and molecular tests, the blood was taken into tubes containing EDTA. For serological tests, the blood was stored in tubes without anticoagulant. All the samples were stored on ice and transported to the laboratory on the same day. Both whole-blood and serum (separated by means of centrifugation at 12,000 g for 10 minutes) were stored separately in microtubes at –20 °C until laboratory testing.

### Collection and identification of ectoparasites

The ectoparasites found during clinical examinations on the animals were collected using forceps and placed in microtubes containing absolute ethanol. These microtubes were then stored at room temperature until the time of identification. Tick and flea taxonomic identifications were performed using dichotomous keys (Linardi & Guimarães, 2000; Barros-Battesti et al., 2006; Dubie et al., 2017).

### Molecular analyses

#### Extraction and amplification of DNA from *Ehrlichia canis*, *Babesia vogeli* and *Hepatozoon* spp.

Total DNA was extracted from 200 μL of canine whole blood using a commercial DNA extraction kit (Invitrogen™ PureLink™ Genomic DNA mini-kit), in accordance with the manufacturer’s instructions. It was eluted in 100 μL of the elution buffer that accompanied the extraction kit. In order to certify the suitability of this DNA extraction protocol, a random sample of 50 blood extracted DNA samples was tested by a PCR assay targeting a ~359-bp fragment of the *cyt-B* mitochondrial gene of vertebrates (Steuber et al., 2005), which confirmed successful extraction.

All canine DNA samples were analyzed by means of two TaqMan real-time PCR protocols: one specific for *E. canis* DNA (Doyle et al., 2005) and the other specific for *B. vogeli* DNA (Peleg et al., 2010). The samples were also tested by means of conventional PCR to detect *Hepatozoon* spp. (Almeida et al., 2012). The sets of primers and probes used in each reaction are described in Table 1. A positive control, from a dog known to be positive for each pathogen tested, and a negative control consisting of water were included in each technique performed, which are described below. Positive control canine DNA samples consisted of *E. canis*-infected blood from the study of Moraes-Filho et al. (2015), *H. canis*-infected spleen from the study of Lopes et al. (2019), and *B. vogeli*-infected blood kindly provided by Prof. Marcos R. André (São Paulo State University, Brazil).

**Table 1:** Primer pairs and probes used in TaqMan real-time PCR assays, for detecting tick-borne agents.

**Target agents (gene)**	**Primers**	**Primer sequences (5’-3’)**	**(Bp)**	**Reference**
*Ehrlichia canis*	Dsb321	TTGCAAAATGATGTCTGAAGATATGAAACA	378	Doyle et al. (2005)
(*dsb* gene)	Dsb671	GCTGCTCCACCAATAAATGTATCYCCTA		
	probe	AGCTAGTGCTGCTTGGGCAACTTTGAGTGAA		
*Babesia vogeli*	*B.c hsp70*-F	GTCATCACTGTGCCTGCGTACT	90	Peleg et al. (2010)
*(hsp70* gene*)*	*B.c hsp70*-R	GCATGACGTTGAGACCGGCAAT		
	probe	AGCGCCAGGCCACCAAGGACGCT		
*Hepatozoon* spp.	HEP2-169	GGTAATTCTAGAGCTAATACATGAGC	574	Almeida et al. (2012)
(18S rRNA)	HEP2-718	ACAATAAAGTAAAAAACAYTTCAAAG		

The real-time PCR for *E. canis* was used to amplify a 378 base pair (bp) fragment of the *dsb* gene encoding a disulfide-forming protein, using the Dsb321 and Dsb671 primers and a species-specific probe, as previously described by Doyle et al. (2005). To detect *B. vogeli*, a 90 bp fragment of the *hsp70* gene was amplified using the *B.c hsp70*-F and *B.c hsp70*-R primers and a species-specific probe, in accordance with the conditions described by Peleg et al. (2010). For these two reactions, data amplification, acquisition and analysis were performed using a multicolor detection system for real-time PCR (7500 Real-Time PCR Systems; Applied BioSystems, Foster City, CA, USA). Samples were considered positive if Ct values were <35. For DNA detection in *Hepatozoon* spp., the primers HEP2 144-169 and HEP2 743 718 were used, which amplified a fragment of about 574 bp of the 18S rRNA gene from *Hepatozoon* spp., as described in the protocol recommended by Almeida et al. (2012).

#### Sequencing

The species of *Hepatozoon* were identified through generating DNA sequences from PCR amplicons. For this, positive samples were purified using ExoSap (USB) and were sequenced in an automated sequencer (model ABI Prism 310 Genetic; Applied Biosystems / Perkin Elmer, California, USA), in accordance with the manufacturer's protocol, and with the same primers as used in the PCR. Sequences were trimmed for quality and edited by using the SeqMan software (Lasergene; DNAstar, Madison, Wis.). The partial sequences obtained were subjected to BLAST analysis (Altschul et al., 1990) to make inferences regarding the closest similarities to the sequences in GenBank.

### Serological analyses

Canine serum samples were tested by means of the Immunofluorescent Antibody Test (IFAT) using crude antigens derived from four Brazilian *Rickettsia* isolates (*Rickettsia rickettsii* strain Taiaçu, *Rickettsia amblyommatis* strain Ac37, *Rickettsia bellii* strain Mogi and *Rickettsia felis* strain Pedreira), as previously described (Labruna et al., 2007). Briefly, the canine serum samples were serially diluted in phosphate-buffered saline (PBS), in twofold increments from 1/64 to 1/2048, and were instilled on glass slides coated with the antigens. A commercial fluorescein isothiocyanate-labeled anti-dog IgG (Sigma^®^, St Louis, MO, USA) was used as a secondary antibody. On each slide, a known non-reactive canine serum (negative control for all antigens tested) and a known reactive canine serum (positive control for all antigens tested) were tested at 1/64 dilution. These sera were from the study of Costa et al. (2017). For each tested serum, the endpoint titer reacting with each *Rickettsia* antigen was determined. Serum that reacted to a *Rickettsia* species with an endpoint titer at least four times higher than the endpoint titers for the other *Rickettsia* species was considered homologous to the first *Rickettsia* species or to a very close genotype, as previously reported (Labruna et al., 2007).

### Statistical analysis

The data were tabulated in LibreOffice version 7.1.0.3 and analyzed in the Statistical Package for Social Sciences (SPSS) for Windows, version 23. In describing the data, absolute and percentage frequencies were used for qualitative variables. In the inferential analysis, the Wald chi-square test or likelihood ratio was used to verify associations between independent and dependent variables. Furthermore, Poisson regression with robust estimation was used to determine the adjusted model, with the respective prevalence ratio (PR) and 95% confidence intervals. For the unadjusted model, variables with p < 0.2 were used. The significance level of the tests was 5%.

## Results

Among the 153 canine blood samples evaluated, 60 (39.2%) yielded signs of infection with at least one of the four pathogens studied. Considering the molecular tests, *B. vogeli* was detected in 23 (15%), *E. canis* in 21 (13.7%) and *Hepatozoon* spp. in 18 (11.8%) of the dogs. Six of the 18 samples that tested positive for *Hepatozoon* spp. were selected for DNA sequencing, in view of the higher intensity of bands obtained through agarose gel electrophoresis. In an analysis on these six sequences using BLAST, all of them showed 100% similarity to *H. canis* detected in domestic dogs in different countries (KJ513193, KJ513198 and KF621083), and also in the northeastern region of Brazil (MG772658). The single haplotype of *H. canis* 18S rRNA partial sequences generated in this study was deposited in GenBank under the accession number OL518910.

Anti-*Rickettsia* spp. antibodies were detected in 20 dogs (13%), with endpoint titers ranging from 64 to 2048 (Table 2). Eleven of these 20 animals presented *R. amblyommatis* as a probable homologous antigen (PHA), for which the endpoint titers were four times greater than the endpoint titers shown for the other four *Rickettsia* species. These 11 animals might have been exposed to *R. amblyommatis* or a very closely related genotype.

**Table 2:** Results from Immunofluorescent Antibody Test (IFAT) against four *Rickettsia* species, among serum samples from dogs in Vila de Jericoacoara, Jijoca de Jericoacoara, Ceará, Brazil, 2020.

Animals	*Rickettsia rickettsii*	Endpoint titers for rickettsial antigens			PHA
		*Rickettsia amblyommatis*	*Rickettsia bellii*	*Rickettsia felis*	
C-08	-	256	-	-	*R. amblyommatis*
C-09	-	2048	-	-	*R. amblyommatis*
C-10	256	256	128	-	-
C-12	256	256	-	-	-
C-24	-	512	-	-	*R. amblyommatis*
C-29	256	512	-	-	-
C-34	512	1024	-	-	-
C-39	-	64	-	-	-
C-40	-	64	-	-	-
C-60	-	64	-	-	-
C-62	-	1024	-	-	*R. amblyommatis*
C-63	-	128	-	-	-
C-64	-	256	-	-	*R. amblyommatis*
C-66	-	256	-	-	*R. amblyommatis*
C-70	-	256	-	-	*R. amblyommatis*
C-88	-	256	-	-	*R. amblyommatis*
C-94	-	1024	-	64	*R. amblyommatis*
C-118	-	256	-	-	*R. amblyommatis*
C-119	-	256	-	-	*R. amblyommatis*
C-134	-	64	-	-	-

Among the 60 infected or exposed animals, 53 were positive for the pathogens investigated by means of molecular detection, among which nine (16.9%) had coinfections in the following combinations: five dogs (55.5%) were coinfected with *E. canis* and *H. canis*, three (33.3%) with *E. canis* and *B. vogeli* and one (11.1%) with *B. vogeli* and *H. canis*.

Parasitism due to ticks was observed in 18 (11.8%) of the dogs. All the ticks collected were identified as *Rhipicephalus sanguineus* sensu lato (s.l.). A total of 49 specimens were found: 19 females, 16 males and 11 nymphs. There was an average of 2.72 ticks/dog, with a range from 1 to 7 ticks per animal. Seven animals that were infected or exposed to tick-borne pathogens (7/60) were infested by ticks. In addition, the flea *Ctenocephalides felis felis* was observed on three (2.0%) dogs. Regarding the responses to the questionnaire about observation of ectoparasites on dogs by their owners, 30 (19.6%) of the responses were positive. The owners indicated that 29 (19%) of the animals were parasitized by ticks and that five (3.3%) were infested with fleas.

The analysis on possible factors associated with positivity for *B. vogeli*, *Hepatozoon* spp. and *E. canis* and seropositivity for *Rickettsia* spp. is shown in Table 3. None of the variables studied was associated with positivity for *E. canis* or with seropositivity for *Rickettsia* spp. (p > 0.05). For infection by *B. vogeli*, the following variables were selected: breed (p = 0.156) and age (p = 0.009). For infection by *Hepatozoon* spp., breed (p = 0.122), age (p = 0.059) and habitation (p = 0.010) were selected. After Poisson regression, only age (< 3 years old) was confirmed to be associated with infection by *B. vogeli* (PR = 2.95; 95% CI 1.23 to 7.07; p = 0.009); and only outdoor habitation (stray dogs) with infection by *Hepatozoon* spp. (PR = 4.0; 95% CI 1.7 to 10.0; p = 0.010).

**Table 3:** Unadjusted analysis on positivity for *Babesia vogeli*, *Ehrlichia canis* and *Hepatozoon* spp. and seropositivity for *Rickettsia* spp., in association with independent factors among dogs in Vila de Jericoacoara, Jijoca de Jericoacora, Ceará, Brazil, 2020.

**Independet Variables**	**N° of dogs**	**Dependent Variables**															
		***Babesia vogeli***				***Ehrlichia canis***				***Hepatozoon* spp.**				***Rickettsia* spp.**			
		**Positive (%)**	**PR**	**p value**		**Positive (%)**	**PR**	**p value**		**Positive (%)**	**PR**	**p value**		**Positive (%)**	**PR**	**p value**	
			**(95%CI)**				**(CI 95%)**				**(CI 95%)**				**(CI 95%)**		
Sex																	
Female	102	17	1.42	0.424	a	11	0.55	0.135	a	11	0.79	0.595	a	9	2.25	0.245	b
		(16.7)	(0.59; 3.37)			(10.8)	(0.25; 1.21)			(10.8)	(0.32; 1.91)			(8.8)	(0.50; 10.03)		
Male	51	6				10				7				2			
		(11.8)				(19.6)				(13.7)				(3.9)			
Breed																	
Mixed	115	20	2.20	0.156	a	3	1,06	0,907	a	16	2.64	0.122	b	9	1.49	0.584	b
		(17.4)	(0.69; 7.00)			(7.9)	(0.42; 2.69)			(13.9)	(0.64; 10.97)			(7.8)	(0.34; 6.58)		
Pure	38	3				20 (17.4)				2				2			
		(7.9)								(5.3)				(5.3)			
Age																	
< 3 years	89	17	2.95	**0.009**	a	11	1.14	0.739	a	12	2,5	0.059	a	3	0.39	0.134	a
		(23.0)	(1.23; 7.07)			(14.9)	(0.52; 2.53)			(16.2)	(0.92; 6.74)			(4.1)	(0.11; 1.41)		
≥ 3 years	62	6				10				5				8			
		(7.8)				-13				(6.5)				(10.4)			
Habitation																	
Stray dog	14	2	0.93	0.917	b	2	1.03	0.966	b	5	4	**0.010**	b	0	- (- ; -)	0,136	b
		(14.3)	(0.24; 3.57)			(14.3)	(0.26; 4.0)			(35.7)	(1,7; 10.0)			(0.0)			
Domiciled	137	21				19				12				11			
		(15.3)				(13.9)				(8.8)				(8.0)			
Street access																	
Yes	113	17	0.90	0.814	a	15	0.96	0.925	b	13	1.04	0.948	b	8	1.27	0.745	b
		(15.0)	(0.39; 2.12)			(13.3)	(0.37; 2.45)			(11.5)	(0.36; 2.98)			(7.1)	(0.28; 5.73)		
No	36	6				5 (13.9)				4				2			
		(16.7)								(11.1)				(5.6)			
Presence of ectoparasites																	
Yes	20	3	1.00	0.996	b	2	0.70	0.590		1	0.39	0.268	b	2	1,48	0.618	b
		(15.0)	(0.33; 3.05)			(10.0)	(0.18; 2.78)			(5.0)	(0.06; 2.78)			(10.0)	(0.34; 6.35)		
No	133	20				19				17				9			
		(15.0)				(14.3)				(12.8)				(6.8)			

a = Wald chi-square; b = Likelihood Ratio.

At the time of physical examination, it was observed that some animals that had been infected or exposed to the pathogens studied presented clinical alterations suggestive of tick-borne diseases, including lymphadenopathy (23/60), fever (8/60), pale mucous membranes (5/60), diarrhea (3/60), anorexia (1/60) and weight loss (1/60). However, there was no significant association between the clinical alterations and infection by *E. canis*, *B. vogeli* or *Hepatozoon* spp., or exposure to *Rickettsia* spp. (p > 0.05).

Table 4 shows analyses on the hematological alterations of the animals studied that were positive for *B. vogeli*, *Hepatozoon* spp. and *E. canis* and seropositive for *Rickettsia* spp. There were significant associations between anemia and infection by *Hepatozoon* spp. (PR = 2.14; 95% CI 1.16 to 3.95; p = 0.036) and between leukopenia and presence of anti-*Rickettsia* antibodies (PR = 8.61; 95% CI 1.60 to 46.21; p = 0.033).

**Table 4:** Analysis on hematological changes associated with positivity for *Babesia vogeli*, *Ehrlichia canis* and *Hepatozoon* spp. and seropositivity for *Rickettsia* spp., among dogs in Vila de Jericoacoara, Jijoca de Jericoacora, Ceará, Brazil, 2020.

**Dependent Variable**	**N° of dogs**	**Independent Variable**															
		***Babesia vogeli***				***Ehrlichia canis***				***Hepatozoon* spp.**				***Rickettsia* spp.**			
		**Positive (%)**	**PR**	**p value**		**Positive (%)**	**PR**	**p value**		**Positive (%)**	**PR**	**p value**		**Positive (%)**	**PR**	**p value**	
			**(CI 95%)**				**(CI 95%)**				**(CI 95%)**				**(CI 95%)**		
Anemia																	
Yes	36	8	1.61	0.168	a	3	0.57	0.260	b	8	2.14	**0.036**	b	2	0.76	0.073	b
		(34.8)	(0.84; 3.09)			(14.3)	(0.19; 1.70)			(44.4)	(1.16; 3.95)			(18.2)	(0.21; 2.75)		
No	117	15				18				10				9			
		(65.2)				(85.7)				(55.6)				(81.2)			
Hyperproteinemia																	
Yes	58	6	0.65	0.205	a	9	1.15	0.615	a	4	0.56	0.144	a	7	1.77	0.073	b
		(26.1)	(0.32; 1.34)			(42.9)	(0.67; 1.98)			(22.2)	(0.23; 1.35)			(63.6)	(1.08; 2.91)		
No	95	17				12				14				4			
		(73.9)				(57.1)				(77.8)				(36.4)			
Leukopenia																	
Yes	5	1	1.41	0.761	b	0	0.00	0.220	b	2	5.00	0.099	b	2	8.61	**0.033**	b
		(4.3)	(0.17; 12.08)			(0.0)	(- ; -)			(11.1)	(0.90; 27.93)			(18.2)	(1.60; 46.21)		
No	148	22				21				16				9			
		(95.7)				(100.0)				(88.9)				(81.2)			
Leukocytosis																	
Yes	47	3	0.39	0.052	a	3	0.43	0.079	a	5	0.89	0.773	b	3	0.88	0.795	b
		(13.0)	(0.13; 1.14)			(14.3)	(0.15; 1.26)			(27.8)	(0.41; 1.96)			(27.3)	(0.33; 2.38)		
No	106	20				18				13				8			
		(87.0)				(85.7)				(72.2)				(72.7)			
Neutrophilia																	
Yes	28	3	0.68	0.464	b	2	0.48	0.231	b	3	0.90	0.847	b	1	0.48	0.376	b
		(13.0)	(0.22; 2.06)			(9.5)	(0.12; 1.89)			(16.7)	(0.30; 2.68)			(9.1)	(0.07; 3.19)		
No	125	20				19				15				10			
		(87.0)				(90.5)				(83.3)				(90.9)			
Neutropenia																	
Yes	1	0	0.00	0.567	b	0	0.00	0.586	b	0	0.00	0.616	b	0	0.00	0.699	b
		(0.0)	(- ; -)			(0.0)	(- ; -)			(0.0)	(- ; -)			(0.0)	(- ; -)		
No	152	23				21				18				11			
		(100.0)				(100.0)				(100.0)				(100.0)			
Monocytosis																	
Yes	30	2	0.40	0.122	b	2	0.45	0.178	b	1	0.26	0.071	b	2	0.92	0.901	b
		(8.7)	(0.10; 1.58)			(9.5)	(0.12; 1.75)			(5.6)	(0.04; 1.79)			(18.2)	(0.25; 3.37)		
No	123	21				19				17				9			
		(91.3)				(90.5)				(94.4)				(81.2)			
Lymphocytosis																	
Yes	30	2	0.40	0.122	b	1	0.22	0.078	b	2	0.54	0.305	b	3	1.43	0.523	b
		(8.7)	(0.10; 1.58)			(4.8)	(0.03; 1.51)			(11.1)	(0.14; 2.06)			(27.3)	(0.52; 3.99)		
No	123	21				20				16				8			
		(91.3)				(95.2)				(88.9)				(72.7)			
Lymphopenia																	
Yes	4	1	1.88	0.599	b	0	0.00	0.274	b	0	0.00	0.314	b	0	0.00	0.437	b
		(4.3)	(0.20; 17.34)			(0.0)	(- ; -)			(0.0)	(- ; -)			(0.0)	(- ; -)		
No	149	22				21				18				11			
		(95.7)				(100.0)				(100.0)				(100.0)			
Eosinophilia																	
Yes	58	5	0.53	0.083	a	5	0.59	0.152	b	3	0.41	0.069	a	4	0.96	0.912	b
		(21.7)	(0.24; 1.19)			(23.8)	(0.27; 13.1)			(16.7)	(0.14; 1.17)			(36.4)	(0.43; 2.15)		
No	95	18				16				15				7			
		(78.3)				(76.2)				(83.3)				(63.6)			
Thrombocytopenia																	
Yes	38	7	1.28	0.500	a	5	0.95	0.907	a	5	1.14	0.761	b	4	1.52	0.377	b
		(30.4)	(0.64; 2.54)			(23.8)	(0.42; 2.16)			(27.8)	(0.51; 2.53)			(36.4)	(0.66; 3.50)		
No	117	16				16				13				7			
		(69.6)				(76.2)				(72.2)				(63.6)			
Thrombocytosis																	
Yes	2	0	0.00	0.418	b	0	0.00	0.441	b	0	0.00	0.478	b	0	0.00	0.584	b
		(0.0)	(- ; -)			(0.0)	(- ; -)			(0.0)	(- ; -)			(0.0)	(- ; -)		
No	151	23				21				18				11			
		(100.0)				(100.0)				(100.0)				(100.0)			

a = Wald chi-square; b = Likelihood Ratio.

## Discussion

This study showed that dogs in Vila de Jericoacoara were infected with *B. vogeli*, *E. canis* and *H. canis* or presented anti-*Rickettsia* spp. antibodies. Although these pathogens had previously been reported infecting dogs in other states in Brazil (Saito et al., 2008; Ramos et al., 2010; Spolidorio et al., 2011; Vieira et al., 2011; Costa et al., 2015; Miranda et al., 2014; Rotondano et al., 2015, 2017; Lopes et al., 2019; Oliveira et al., 2020), data relating to Ceará were very scarce, and were mainly from the coastal region. Recently, *R. rickettsii, R. amblyommatis* and *E. canis* were reported infecting dogs in the National Forest (FLONA) of Araripe-Apodi, in the municipality of Crato, state of Ceará (Oliveira et al., 2020). However, there had not been any previous reports of *H. canis* and *B. vogeli* in dogs in this state.

The occurrence of infection by tick-borne pathogens in dogs observed in this study reflected problems in sanitary management in the region studied. Certain factors may have favored transmission of ectoparasites among animals. Figueredo et al. (2017) assessed exposure to vector-borne pathogens among privately owned dogs that were living in four Brazilian cities, in two states (Pernambuco and Minas Gerais) and in the Federal District. Overall, 69.3% of the dogs were positive for at least one of the pathogens tested (*Anaplasma* spp., *Ehrlichia* spp., *Babesia* spp*., Leishmania* spp. and *Dirofilaria immitis*), and 66.8% of them were positive for two or more pathogens. According to these authors, there is a need to establish a relationship between the socioeconomic status of the owners and the level of exposure to ectoparasites and the pathogens that they transmit. In our study, the dog owners selected had low income levels.

In the present study, *R. sanguineus* s.l. was the only tick species found. Interestingly, none of the known tick vectors for the SFG in Brazil were detected on these animals. In the municipality of Patos, state of Paraíba, also in northeastern Brazil, *R. sanguineus* s.l. was also the only species found among the dogs. Ticks of the genus *Amblyomma* were not detected (Tanikawa et al., 2013). According to these authors, environmental factors such as the semiarid climate and the typical xeric forest of the Caatinga biome may have had an association with the absence of tick vectors of the genus *Amblyomma* in the study region. On the other hand, Oliveira et al. (2020) proved that dogs in the FLONA of Araripe-Apodi were infested with *R. sanguineus* s.l., *Amblyomma parvum* and *C. felis felis*; and that specimens of *A. parvum* and *C. felis felis* were infected with *Rickettsia* spp. Parasitism by other tick species in dogs in Vila de Jericoacoara should not be ruled out, given the presence of anti-*R. amblyommatis* antibodies in the animals studied. Moreover, this village is surrounded by the National Park of Jericoacoara, which forms a domestic-wild animal interface with large diversity of animals and ectoparasite species.

*Rickettsia amblyommatis* has been reported infecting *Amblyomma auricularium*, *A. parvum* (Saraiva et al., 2013; Lugarini et al., 2015; Oliveira et al., 2020), *Amblyomma longirostre* (Ogrzewalska et al., 2011; Lugarini et al., 2015; McIntosh et al., 2015), *Amblyomma cajennense* sensu stricto (ss) (Costa et al., 2017), *Amblyomma pseudoconcolor* (Silva et al., 2018) and *Amblyomma varium* (Lugarini et al., 2015) in northeastern Brazil. Although the pathogenicity of *R. amblyommatis* to humans has not yet been proven, some cases of Rocky Mountain spotted fever in the United States may have been caused by this bacterium (Apperson et al., 2008). Studies have demonstrated that dogs were naturally infected with *R. amblyommatis* in northeastern Brazil (Costa et al., 2017) and in the United States (Barrett et al., 2014). Saraiva et al. (2013) confirmed the vector competence of *A. auricularium* for *R. amblyommatis*. Considering the exposure to *R. amblyommatis* among the dogs studied here, it can be suggested that ticks of the genus *Amblyomma* were present in Vila de Jericoacoara. Furthermore, *C. felis felis* fleas were previously found to be infected with *R. felis* in Ceará (Oliveira et al., 2020). However, the dogs tested in the present study did not show titers that would correspond to exposure to the species *R. felis*, although *C. felis felis* fleas were found on three animals.

Our study was the first to confirm the occurrence of *B. vogeli* in the state of Ceará through molecular tests. The results found demonstrated a high rate of occurrence, compared with other recent reports in northeastern Brazil. The molecular prevalence of canine babesiosis in this region of Brazil has ranged from 0.9% to 10% (Silva et al., 2012; Rotondano et al., 2015; Costa et al., 2015; Silva et al., 2016; Braga et al., 2019).

The occurrence of infection by *E. canis* in the canine population of Vila de Jericoacoara was similar to that found in other studies conducted in the northeastern region of Brazil. Rotondano et al. (2017) found the molecular occurrence of *E. canis* 8.9% among dogs in an urban area in the state of Paraíba. In this region of Brazil, the molecular ocurrence of *E. canis* infection has ranged from 1.7% to 25% (Tanikawa et al., 2013; Costa et al., 2015; Rotondano et al., 2015, 2017; Dantas-Torres et al., 2018).

The occurrence of infection by *Hepatozoon* spp. of 11.8% (confirmed as *H. canis* in six dogs) among the dogs in Vila de Jericoacoara corroborated previous data on the circulation of this parasite in northeastern Brazil. The rates have ranged from 0.49% in Pernambuco to 10% in Rio Grande do Norte (Ramos et al., 2010; Bernardino et al., 2016; Lopes et al., 2019). The occurrence rate can range from 8.6% to 100% in the southeastern region (O'Dwyer et al., 2001; Mundim et al., 2008; Spolidorio et al., 2009; Miranda et al., 2014) and from 3.6% to 73% in the central-western region (Paludo et al., 2003; Mundim et al., 2008; Ramos et al., 2015; Melo et al., 2016; Sousa et al., 2017). In addition, cases of infection by *Hepatozoon* spp. detected through molecular analyses were reported in the southern region (Lasta et al., 2009; Malheiros et al., 2016; Mongruel et al., 2018) and in the northern region (Gomes et al., 2016).

The molecular tests revealed different combinations of coinfections among the dogs in Vila de Jericoacoara. Coinfections have also been reported in other studies (Santos et al., 2009; Ramos et al., 2010; Spolidorio et al., 2011) and have occurred because *B. vogeli*, *H. canis* and *E. canis* are transmitted by the same vector; i.e. the brown tick *R. sanguineus* s.l.. This result serves as a warning with regard to the consequences of coinfection, such as worsening of clinical abnormalities, and the importance of correct diagnosis, in order to be able to indicate the appropriate treatment (Rojas et al., 2014).

Our results indicated that the occurrence rate of infection by *B. vogeli* was higher among animals that were less than three years old, compared to older animals. This can be explained by the immaturity of the humoral immune system in young dogs. According to Bashir et al. (2009), these animals may not have the full capacity to produce antibodies against pathogens, although the cellular immune response also plays an important role in the immune response against this protozoan. Similar results were found by Rotondano et al. (2015). These authors observed that newly weaned young dogs were more susceptible to disease due to the stress of adapting to food and the environment. Our results also corroborate those of Paulino et al. (2018), who found that animals under five years of age were more likely to test positive for *B. vogeli* DNA.

The type of habitation of the animals living in Vila de Jericoacoara was associated with positivity for *H. canis*. The occurrence rate for infection by this pathogen was higher among stray dogs than among domiciled dogs. Corroborating our findings, Aktas et al. (2015) demonstrated that stray and shelter dogs showed significantly higher prevalence of *H. canis* infection, compared with pet dogs. We can hypothesize that these dogs are more prone to infection due to greater exposure to the vector and lack of veterinary care.

In our study, no associations between clinical alterations in the dogs and infection or exposure to the pathogens studied were demonstrated. According to Mundim et al. (2008), the clinical presentation of vector-borne diseases varies according to the level of parasitemia and the animal's immune status. Moreover, 48.33% of the infected or exposed animals studied here did not present any clinical signs suggestive of tick-borne diseases, and 60.21% of the uninfected or unexposed animals presented lymphadenopathy, weight loss, anorexia, vomiting, diarrhea or fever.

It was observed in Vila de Jericoacoara that anemia was associated with infection by *H. canis*. Some changes to animals positive for *H. canis* had been previously described, including anemia, leukocytosis with neutrophilia, lymphopenia, monocytosis and thrombocytopenia (Paludo et al., 2003; Aguiar et al., 2004; Antunes et al., 2015; Mongruel et al., 2018). Regarding white blood cells, it was observed that leukopenia was associated with the presence of rickettsial antibodies. Alterations such as anemia, thrombocytopenia and moderate initial leukopenia, followed by leukocytosis, have been described in animals positive for *Rickettsia* spp. (Keenan et al., 1977a, b; Breitschwerdt et al., 1988; Comer, 1991). However, the hematological alterations presented by infected animals can also be caused by other pathogens and by exposure to allergens. Furthermore, our results showed that 16.6% of the infected or exposed animals had a normal hematological profile.

## Conclusions

In this study, circulation of *B. vogeli, H. canis* and *E. canis* in dogs in the coastal region of the state of Ceará, northeastern Brazil, was proved. It was noteworthy that *Rickettsia* spp., mainly represented by *R. amblyommatis*, was also circulating among dogs in Vila de Jericoacora. As far as we know, this study provided the first evidence on circulation of these pathogens among dogs in the region analyzed. Canine active infection by *E. canis* and *B. vogeli* indicates environmental contamination by the tick vector, *R. sanguineus* s.l., which ensures occurrences of primary infection in young dogs. This study may help to elucidate the natural history of tick-borne diseases and serve as a warning regarding the need to intensify ectoparasite control among dogs, considering that they may be infected with these agents or with others that were not evaluated in this report.
